# Synaptic Metaplasticity Underlies Tetanic Potentiation in *Lymnaea*: A Novel Paradigm

**DOI:** 10.1371/journal.pone.0078056

**Published:** 2013-10-29

**Authors:** Anita Mehta, Jean-Marc Luck, Collin C. Luk, Naweed I. Syed

**Affiliations:** 1 S. N. Bose National Centre for Basic Sciences, Calcutta, India; 2 Institut de Physique Théorique, CEA Saclay and CNRS URA 2306, Gif-sur-Yvette, France; 3 Hotchkiss Brain Institute, Faculty of Medicine, University of Calgary, Alberta, Canada; Western University of Health Sciences, United States of America

## Abstract

We present a mathematical model that explains and interprets a novel form of short-term potentiation, which was found to be use-, but not time-dependent, in experiments done on *Lymnaea* neurons. The high degree of potentiation is explained using a model of synaptic metaplasticity, while the use-dependence (which is critically reliant on the presence of kinase in the experiment) is explained using a model of a stochastic and bistable biological switch.

## Introduction

All brain functions, ranging from simple reflexes to complex motor patterns, learning and memory, rely upon synaptic transmission between neurons through specialized structures termed synapses. These synaptic connections are, however, not static in nature; in fact, they exhibit a high degree of synaptic plasticity, enabling a network to generate behaviorally relevant and functionally meaningful outputs. These changes in synaptic strength can either be short- or long-term, and may form the basis for both short- and long-term memory, respectively.

Synaptic plasticity is neither restricted to any select group of neurons nor to a particular species – rather it is a universal trademark of all neurons that have been investigated to date. When a nervous system is unable to exhibit modulatory changes associated with short- and long-term synaptic plasticity, it is rendered dysfunctional. Therefore, defining the mechanisms underlying synaptic plasticity is not only pivotal for our understanding of how the brain functions but also for managing the behavioral, learning, memory and cognitive defects that are met in clinical practice. However, despite recent advances in our understanding of various modes of neuronal communication, the cellular and molecular mechanisms underlying synaptic plasticity remain poorly defined. Moreover, the data generated from experimental studies has often been inadequate to garner mathematical modeling predictions that may aid future research in this area, or to provide insights into the mechanisms of synaptic plasticity. This field could however benefit from a paradigm shift where modeling approaches could be used to predict elements of synaptic plasticity and to facilitate future research in the area of metaplasticity.

In a recent study [1], two of the authors of this paper observed a form of short-term potentiation induced by tetanic stimulation, whose time-frame exceeded conventional forms of short-term potentiation. While the induction of potentiation was similar to previous forms of short-term potentiation, the time frame of the potentiated response was characteristic of long-term potentiation (∼ 5 hrs).

**Figure 1:**
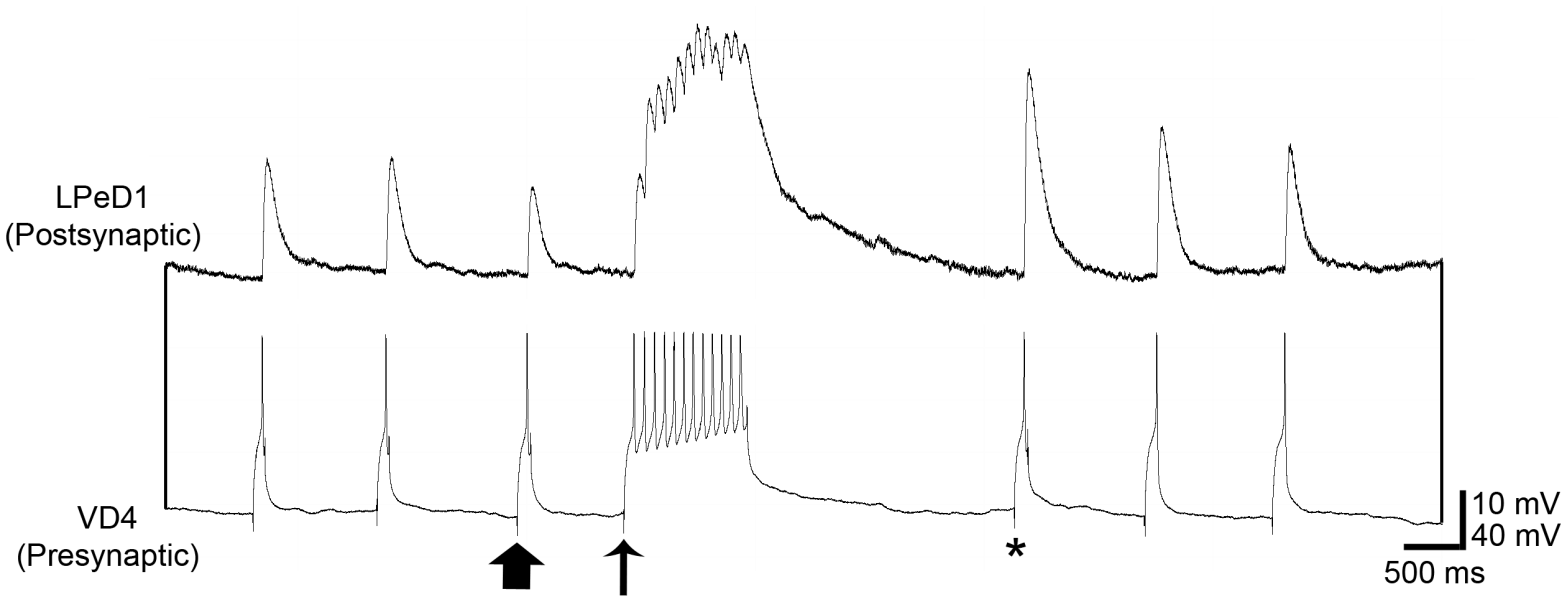
A representative electrophysiology trace showing the potentiation of the VD4/LPeD1 synapse following tetanic stimulation and its subsequent depotentiation. A tetanic stimulation of presynaptic neuron VD4 (at thin arrow) results in a compound excitatory postsynaptic potential (EPSP) in postsynaptic neuron LPeD1. A subsequently triggered action potential in VD4 (asterisk) results in an EPSP of greater amplitude than pre-tetanic stimulation (thick arrow).

More specifically, using well-defined, excitatory cholinergic synapses between *Lymnaea* pre- and postsynaptic neurons, specifically visceral dorsal 4 (VD4) and left pedal dorsal 1 (LPeD1-Excitatory), they provided evidence for a novel form of short-term potentiation, which was use-, but not time-dependent. They found that following a tetanic stimulation (∼ 10 Hz) in the presynaptic neuron with a minimum of seven action potentials, the synapse became potentiated, whereby a subsequent action potential triggered in the presynaptic neuron resulted in an enhanced postsynaptic potential (see Figure 1). Further, if an inducing tetanic stimulation was activated but a subsequent action potential was not triggered, the synapse was shown to remain potentiated for as long as 5 hours. However, once this action potential was triggered, the authors found that the synaptic strength rapidly returned to baseline levels. It was also shown that this form of synaptic plasticity relied on the presynaptic neuron, and required pre- but not postsynaptic Ca^2+^/calmodulin dependent kinase II (CaMKII) activity. Hence, this form of potentiation shares induction and de-potentiation characteristics similar to other forms of short-term potentiation, but exhibits a time-frame analogous to that of long-term potentiation. The model of metaplastic synapses [2] reviewed below allows us to reproduce these long timescales, and forms the basis of our explanation of the experimental results reported in [1].

In the model of metaplastic synapses, incoming signals are stored as memories at progressively deeper levels of a synapse, leading to a clear temporal separation between long- and short-term memory. The upper levels are more vulnerable to ‘noise’, i.e., the regular influx of (usually irrelevant) information which is responsible for the phenomenon of forgetting; only short-term memories can be stored here. (For a review of noise in neural systems, see [3]). Deeper synaptic levels are more protected from this noise, and thus able to retain the memory of applied signals for a much longer time. Drawing on these structural ideas first proposed by Fusi et al. [4], the model of [2] provides a theoretical framework for the *dynamics* of signal propagation within the metaplastic synapse. It suggests that random signals are typically stored in the upper levels of the synapse for relatively short times, and then lost to noise: non-random signals, on the other hand, are stored as long-term memories in the deepest synaptic levels and forgotten much more slowly.

The link between this theory and the experiment reported in [1] relies on the fact that the output signal in the latter was *amplified* after a process of tetanic stimulation. This suggested the following scenario: the initial action potentials, interpreted as a non-random signal, cumulatively built up a long-term memory of the signal in the deepest synaptic levels. The synapse dynamics were then frozen so that further discharge was prevented. When a further action potential was applied, the synaptic dynamics restarted (‘use’-dependence): the release of the accumulated memory from the deepest levels of the synapse constituted the observed enhancement of the output signal described in [1]. While this enhancement is plausibly accounted for by the model of metaplastic synapses [2], the explanation of the freezing of the synaptic dynamics and its subsequent use-dependence needs the introduction of a biological switch. The stochastic and bistable switch presented in this paper meets this need, and models the role of kinase (CaMKII) in the actual experiment [1].

In the following, we first review the basics of the model of a metaplastic synapse [2]. We then provide a full theoretical framework for the explanation of the experimental results, with an emphasis on the dynamics of the biological switch. We close by discussing our results.

## Results

### A Model of a Metaplastic Synapse

If synapses are highly plastic, signals are quickly stored: however, high plasticity also means that more and more signals are stored, generating enough ‘noise’ so that ‘memories’ of earlier signals soon become irretrievable. Clearly, this is at variance with the fact that long-term memories are ubiquitous in human experience; it was to resolve this paradox that models of metaplastic synapses were formulated [4]. The idea behind such models was that the introduction of ‘hidden states’ for a synapse would enable the delinking of memory lifetimes from instantaneous signal response: while maintaining quick learning, this mechanism would also be able to allow slow forgetting. This was implemented by the storage of memories at different ‘levels’: the relaxation times for the memories increased as a function of depth. This hierarchy of time scales models the phenomenon of metaplasticity [5], [6].

In [2] these ideas were put into a new framework, with the dynamics of signal processing playing a central role. Also, two different internal synaptic structures were investigated: the first (Model I) was very similar to Fusi’s original model [4], while the second (Model II) had a different architecture. In the following, we focus on the second model. We start by reviewing its essential features.

**Figure 2:**
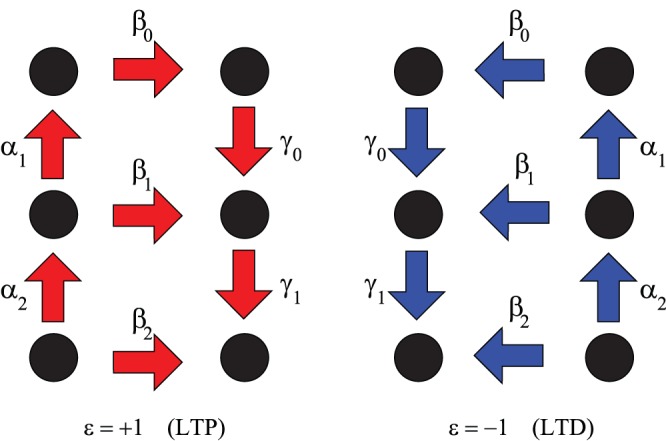
Schematic representation of the model of the internal synaptic structure. Arrows denote possible transitions in the presence of a potentiating pulse (PP, 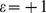, left panel) and of a depressing pulse (DP, 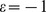, right panel). Corresponding transition probabilities are indicated. In each panel, the left (resp. right) column corresponds to the − (resp. +) state. The model studied in this work is actually infinitely deep. After [2].

The dynamics of the model are defined in Figure 2. At every discrete time step 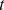, the synapse is subjected either to a potentiating pulse (PP) (encoded as 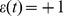) or to a depressing pulse (DP) (encoded as 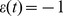), where 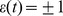 is the instantaneous value of the input signal at time 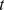. There are three outcomes of the application of a PP signal:

- 3 pt If the synapse is in its − state at depth 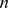, it may climb one level 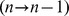 with probability 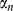.
- 3 pt If it is in its − state at depth 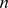, it may alternatively cross over to the + state *at the same level* with probability 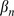.
- 3 pt If it is already in its + state at depth 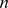, it may fall one level 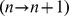 with probability 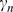.

The *level-resolved output signal* of level 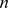 at time 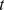:
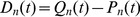
(1)and the *total output signal* at time 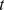:
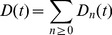
(2)can be expressed in terms of the probabilities 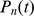 (resp. 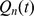) for the synapse to be in the − state (resp. in the + state) at level 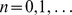 at time 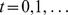 The dynamical equations obeyed by the latter probabilities are reviewed in the Methods section, along with other details for the mathematically inclined reader.

**Figure 3:**
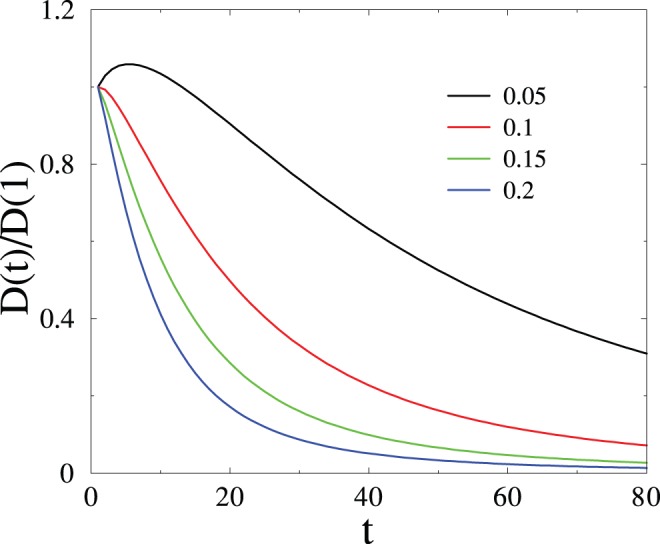
Plot of the reduced output signal 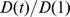 after a single PP input signal, against time 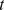, for several 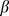. After [2].

Before any meaningful signal is applied, the synapse is assumed to be in its default state. The latter state is defined as the stationary state reached by the synapse if subjected to a long random input signal. It is described in detail in the Methods section (see equations (16) to (21)). When a single potentiating pulse signal is applied at time 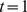 (that is, 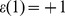) to the synapse in its default state, the synapse will get polarized in response, and thus ‘learn’ the signal. Later on, under the influence of a random input signal for times 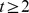, it will ‘forget’ the PP signal, and return to its default state. Figure 3 shows plots of the reduced output signal 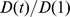 against time 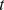 for several values of the control parameter 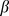. All subsequent figures refer to the parameter values 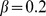, 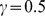, and 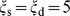 (see Methods section). From here on, we will refer to times where the synapse is subjected to a significant signal (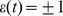) as *learning phases*, and to times where the synapse is subjected to random input (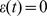) as *forgetting phases*.

The late stages of the forgetting process are characterized by a universal power-law decay of the output signal:
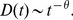
(3)


This is known as *power-law forgetting* [7]–[9]. The forgetting exponent
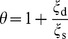
(4)is always larger than unity and depends on the ratio of the dynamical and static lengths 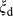 and 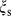. If the synapse were finite rather than infinite, and consisted of 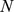 levels, the power-law decay (3) would be exponentially cut off at a time
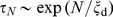
(5)which grows exponentially fast with the ratio of the number 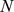 of levels to the dynamical length 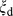.

**Figure 4:**
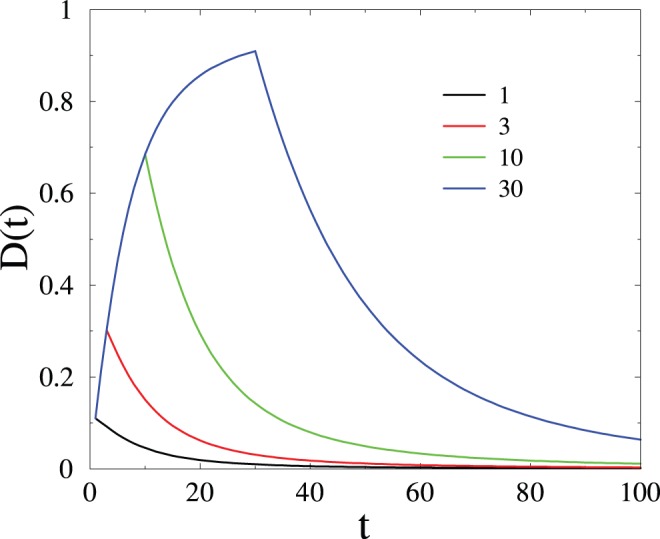
Plot of the output signal 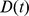 against time 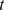, for several durations 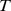 of the LTP signal. After [2].

We now describe the effect of a sustained input of potentiating pulses lasting for 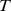 consecutive time steps (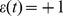 for 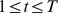) on the model synapse: in the following, this will be referred to as a long-term potentiating (LTP) signal. The synapse is again assumed to be initially in its default state. The learning and forgetting processes are qualitatively similar to the PP case described above, while novel qualitative features emerge when the duration of the signal is long enough (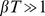). In this regime, the synapse gets almost totally polarized under the persistent action of the input signal. This saturation phenomenon is illustrated in Figure 4, which shows the output signal 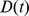 for several durations 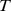 of the LTP signal.

The synapse slowly builds up a long-term memory in the presence of a long enough LTP signal, as the memorized signal moves to deeper and deeper levels. At the end of the learning phase (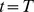), the polarisation profile will have the form of a sharply peaked traveling wave, around a typical depth which grows according to the logarithmic law
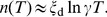
(6)


The total output signal then decays according to the universal power law (3), irrespective of the duration of the learning phase, driving home the universality of power-law forgetting.

The above model provides a mechanism for the long-term memory storage due to use dependence in the *Lymnaea* synapse examined in [1]. With the tetanic stimulation acting as an LTP signal, this model shows that the synapse becomes fully polarized, the memory of the stimulating pulses being ‘stored’ in a deep level. However, another concept is needed to model the subsequent freezing of the synaptic dynamics: a major clue is provided by the fact that it is suggested in [1] that the activation of CaMKII in the presynaptic cell acts like a ‘molecular switch’. Accordingly, we present a theoretical model of a *stochastic and bistable* switch in the following section.

### The Effect of Use-dependent Synaptic Potentiation: Coupling to a Stochastic and Bistable Switch

In this section, we present a model of a biological switch to describe the role of CaMKII in the experiment reported in [1]. The switch can exist in two states, ‘on’ or ‘off’, which we label by the binary variable 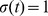 or 0. Since natural processes are usually stochastic rather than deterministic, we incorporate this by postulating that the switch is on with probability 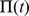, and off with the complementary probability 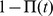. Thus:
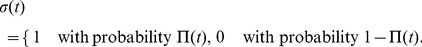
(7)


The main effect of this switch is to freeze the synaptic dynamics after adequate potentiation: we accordingly refer to the probability 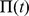 as the *freezing probability*. Thus:

- 3 pt If the switch is *off* (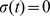), the synapse evolves as usual. This occurs with probability 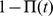.
- 3 pt If the switch is *on* (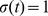), the forgetting process (‘discharge’) is frozen. This occurs with probability 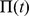.

More precisely, the synapse learns via (12)-(13) and forgets via (18) when the switch is off. When the switch is on, the synapse still learns via (12)-(13), but it does not forget at all.

In the experiment, a minimum of seven action potentials is needed to activate the switch and freeze the dynamics: this suggests that the switch somehow responds to the saturation of the synaptic capacity, so that freezing *never* sets in for less than this number of action potentials. Also, a further tetanic pulse after a period of quietude is needed to restart the synaptic dynamics. We design the evolution of the freezing probability 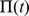 accordingly:

- 3 pt If the synapse is within a learning phase (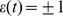 and 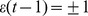), the freezing probability evolves according to the quadratic rule

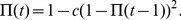
(8)This rule ensures that the freezing probability increases with the number of action potentials applied, saturating quickly to 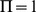 and freezing the synaptic dynamics after a threshold number of these is reached. It is desirable that the fixed point of 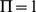 is superstable (see below), and the quadratic law (8) is the simplest non-linear law which ensures this.

- 3 pt If the synapse is in a forgetting phase (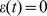), the freezing probability is itself frozen to its value inherited from the past:

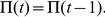
(9)This rule ensures that once frozen, the dynamics stay frozen and that the synapse stays potentiated, until a further action potential is applied.

- 3 pt If the synapse is at the first step of a learning phase (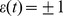 but 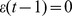), the freezing probability is instantly reset to

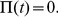
(10)This ensures that the synaptic dynamics restart, as soon as an action potential is applied for the first time following a period of forgetting.

Note that there is a ‘soft’ threshold in the experiment for the switch to kick in, in that a *minimum* of seven action potentials is required; as reported in [1], this phenomenon was usually seen to occur across a range of 7 to 14 action potentials. The freezing probability which models the switch dynamics needs to incorporate this soft threshold, for which superstability of the fixed point 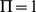 is desirable. This ensures that at the end of a sustained LTP signal of duration 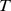, 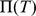 converges very rapidly to unity (more rapidly than exponentially), as soon as 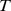 exceeds a characteristic time 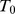, defined operationally by 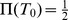. The value of the characteristic time fixes the parameter 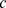. We have then
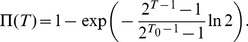
(11)


**Figure 5:**
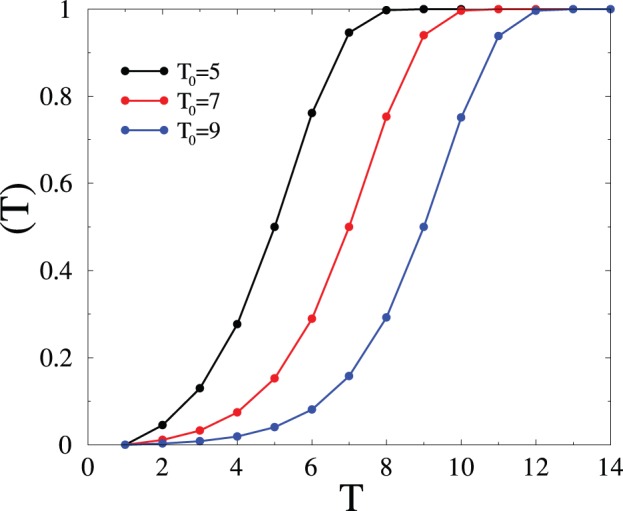
Plot of the freezing probability 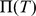 at the end of an LTP signal, against its duration 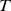, for three values of the characteristic time 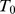. The threshold for freezing is least for the black curve (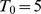), and most for the blue (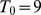): while, say, 8 action potentials will definitely cause the onset of freezing for the black curve (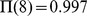), it will only rarely do so for the blue one (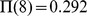).

Figure 5 shows a plot of the freezing probability 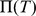 at the end of an LTP signal of duration 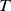 for several values of the characteristic time 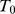 in the realistic range of 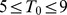. In practice, as soon as 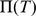 is appreciably large, the switch kicks in stochastically – and this can occur, as shown in Figure 5, over a range of signal durations.

We now commence a global interpretation of the experiment. The synapse is assumed to be initially in its default state, with 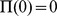. It is then subjected to a sustained LTP signal of duration 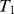 (i.e., the application of 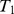 action potentials), and to a single action potential at a much later time (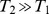). The synapse is subjected to a random input at all the other instants of time (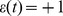 for 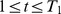 and for 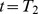, else 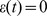).

In the regime where the number of action potentials 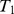 of the initial signal is larger than the characteristic time 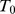 of the switch, the freezing probability 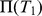 at the end of the LTP period is very high, i.e., very close to unity (see Figure 5). During this learning phase, the output signal 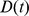 grows progressively from 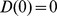 to a large value 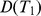. The high value of 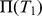 at the end of this phase typically freezes the synaptic dynamics, ensuring that this enhanced output signal is not discharged. When the next action potential is applied at time 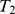, the switch is turned off, and the synapse then relaxes via the full discharge of the stored, enhanced output signal.

**Figure 6:**
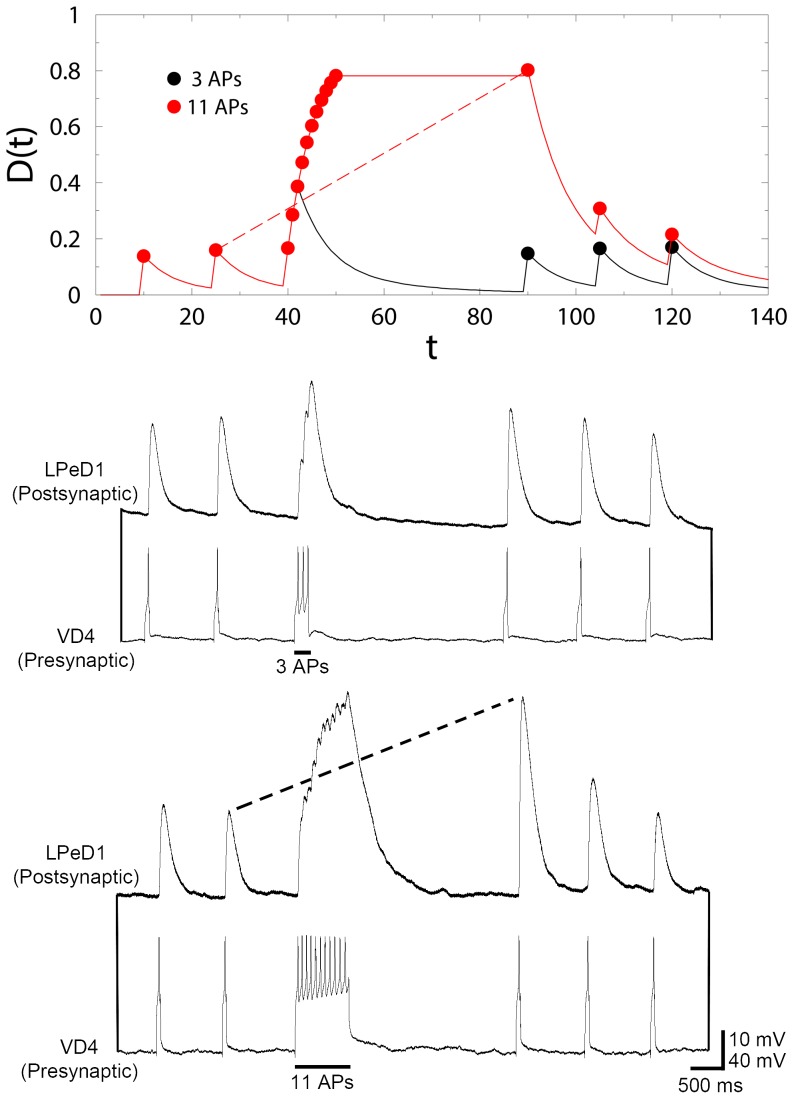
An integrative figure showing the good qualitative agreement of theory and experiment. The two lower panels show sharp-electrode electrophysiology recordings of a VD4/LPeD1 synaptic pair. During the induction phase, three action potentials were triggered at ∼ 10 Hz. The synapse was allowed to remain quiescent for ∼ 5 s and when a subsequent action potential was triggered, the amplitude of the postsynaptic potential was similar to pre-tetanic stimulation. However, when eleven action potentials were triggered at ∼ 10 Hz, a potentiated response was observed after the same quiescent period of ∼ 5 s after stimulation. The upper panel shows the predictions of the theoretical model, also for three and eleven action potentials.

We now compare theory with experiment. Figure 6 shows a comparison between our theoretical predictions (upper panel) with sharp-electrode electrophysiology recordings of a VD4/LPeD1 synaptic pair (two lower panels). The theoretical prediction is meant to describe the average over many ensembles, while the experimental data are assumed to be typical. Since the experimental averages are well-behaved rather than subject to large fluctuations [1], a typical experimental output is representative of its average. On the theoretical side, the time unit for the discrete updates is consistently chosen to be the time interval between two successive applied APs, i.e., 100 ms. The black theoretical curve corresponds to 3 APs triggered during tetanic stimulation, which are insufficient to result in potentiation of a subsequent excitatory postsynaptic potential (EPSP) in the LPeD1 neuron (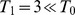, and so the switch remains off). The red theoretical curve corresponds to 11 APs, resulting in a potentiated response (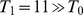, so the switch is turned on and the synapse is frozen). The predicted pattern of peak heights (symbols) is rather robust, i.e., insensitive to model parameters, and provides a good overall description of the experimental recordings.

**Figure 7:**
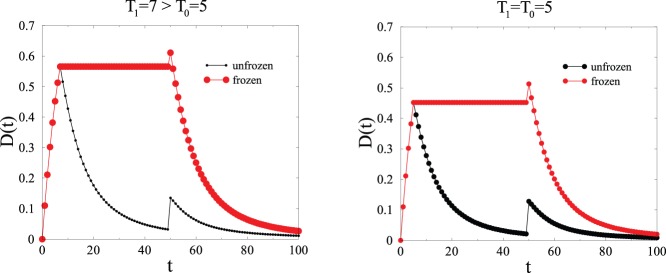
Plot of the two possible kinds of output signals 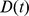 generated by the protocol described in the text, against time 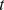 (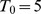, 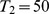). Symbol sizes are proportional to the probabilities of each kind of behavior, i.e., 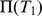 for the frozen one and 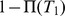 for the unfrozen one. Left: 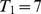 is larger than 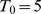, so that 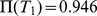 is very high. Right: 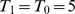, so that 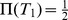.

The last part of this section reflects the ‘bistability’ of the switch, i.e., its stability in one of two states. If the duration 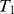 of the initial LTP signal is comparable to the characteristic time 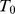, the synapse may exhibit two types of temporal behavior. It may behave as above for 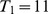, i.e., stay frozen until the subsequent action potential unfreezes it at time 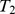. This occurs with probability 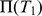 (see Figure 5). The synapse may also remain unfrozen, so that the output signal decays almost entirely before the subsequent action potential sets in. This occurs with the complementary probability 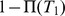. This bistable behavior is illustrated in Figure 7. We see from this figure that the difference between 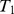 and 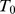 provides a tunable parameter which determines how frequently the phenomenon described in the experiment in [1] is observed: in the upper panel the parameters are such that it is almost always observed, whereas in the lower panel it makes a much more random appearance. This parametrisation is useful, since it can form the basis of future experiments. If we assume that the characteristic time 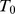 is, say, inversely proportional to the concentration of kinase, this suggests that a threshold concentration can be identified for which a given number of action potentials will cause the short-term potentiation of the synapse.

## Discussion

Our theoretical model of a biological switch coupled to a metaplastic synapse is able to provide an interesting framework for the interpretation of the experimental results of [1]. This novel form of synaptic potentiation exhibits characteristics of short-term potentiation, but also exhibits characteristics of long-term potentiation based on its time frame. While the enhanced output signal observed in the latter is attributable to long-term potentiation of the synapse via tetanic stimulation, the use-dependence of the synapse is explainable by the biological switch.

We start with the depotentiated state of the synapse, where, experimentally, single action potentials triggered in VD4 elicit EPSP in LPeD1 of a non-potentiated amplitude. The application of repeated action potentials in the pre-synaptic cell however builds up long-term memory in the synapse, i.e., a potentiated EPSP in LPeD1. Meanwhile, during the tetanic stimulation, the freezing probability of the molecular switch (CaMKII) evolves according to (8) in response to the growing saturation of the synapse. If the number of action potentials is large enough, the switch is activated and synaptic dynamics are fully frozen. Synaptic dynamics are only restarted when the switch is turned off: this happens when the synapse is next ‘used’, and leads to the observed rapid decay back to a non-potentiated amplitude in LPeD1. Thus, the critical dependence of the onset of the decay on synapse *use*, rather than the *time* elapsed after priming, is explained by the model presented in this paper.

It is useful to discuss some of the key assumptions made here before concluding. The level of description of our model is sufficiently abstract that it does not claim to replicate intricate biological detail; its description is limited to probing the unusually long times of use-dependent synaptic potentiation in the Lymnaea synapse. From this point of view, the use of a model of long-term memory in metaplastic synapses is perfectly appropriate. This model embodies the idea that forgetting takes place via the exposure of the multi-level synapse to noise, from which the lowest synaptic levels are protected. This noise could refer to fluctuations in neural/synaptic activity or to background signals; what is clear is that it is ubiquitous and unavoidable. The model of a biological switch which we introduce depends both on the presynaptic cell and on the state of the synapse: it is turned on when the synapse is saturated in response to a series of action potentials, leading to the freezing of synaptic dynamics. It is turned off when the synapse is next ‘used’, i.e an activation potential is applied after an inert period. An enhanced output signal results, because of the discharge of accumulated memory at the synapse. The turning on and off of the switch is stochastic, rather than deterministic, given the nature of most natural processes: and it is bistable, since it can lead to two kinds of temporal behaviour at the synapse (with different probabilities, of course). Finally, since the experimental recordings do not show strong fluctuations [1], the output of a typical experiment can be meaningfully compared with the average output signal given by theory.

While the intact brain is composed of a significantly greater number of synapses with far more intricate connectivity patterns, the current model has taken a reductionist approach to understanding synaptic plasticity at the level of a single synapse. While previous forms of synaptic potentiation have been modeled, this novel form of use-dependent synaptic potentiation has not. Therefore, this study may not only be an important step in developing more complex models composed of multiple synapses, but also be important in guiding further research in understanding neuronal network function in intact *Lymnaea* brain.

## Methods

The basic quantities used to describe the state of the synapse are the probabilities 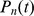 (resp. 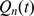) for the synapse to be in the − state (resp. in the + state) at level 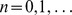 at time 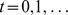 These probabilities obey the following coupled linear equations, whose form is characteristic of Markov chains [10]:

• 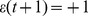 (see Figure 2, left):
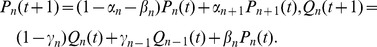
(12)


• 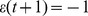 (see Figure 2, right):
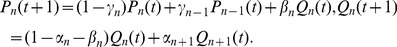
(13)The transition probabilities of the model are assumed to decay exponentially with level depth 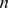:
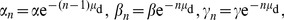
(14)where the *dynamical length*

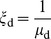
(15)measures the number of fast levels at the top of the synapse.

The *default state* of the synapse is defined as its stationary state in the presence of a random input signal, defined by choosing at each time step
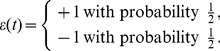
(16)


The dynamics of the synapse subjected to such a random input, referred to as a ‘white-noise’ random input in [2], is defined by averaging the linear dynamical equations (12)-(13) over both instances of 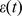 at each time step. The resulting average dynamics is formally labeled as 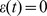. It has a simpler expression in terms of the sums and differences
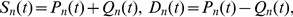
(17)namely
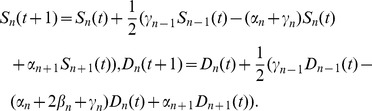
(18)The default state of the synapse, defined as the stationary state of the above average dynamics, is characterized by the probabilities
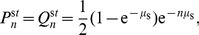
(19)with
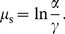
(20)The default state is appropriately featureless and unpolarized, as it should be for a symmetric synapse. The corresponding *static length*

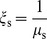
(21)gives a measure of the effective number of occupied levels in the default state.
