# Correction: Synaptic Metaplasticity Underlies Tetanic Potentiation in *Lymnaea*: A Novel Paradigm

**DOI:** 10.1371/annotation/230cca90-58e9-4aa1-b6b2-a1d744524fbd

**Published:** 2014-01-21

**Authors:** Anita Mehta, Jean-Marc Luck, Collin C. Luk, Naweed I. Syed

The publisher apologizes for the following errors. In the Results section, line breaks were inadvertently removed in Equations 7, 12, 13, and 18. In the "A Model of a Metaplastic Synapse" section and the "The Effect of Use-dependent Synaptic Potentiation: Coupling to a Stochastic and Bistable Switch" section, "3 pt" should not appear after the bullets.

Please see the correct equation 7 here: 


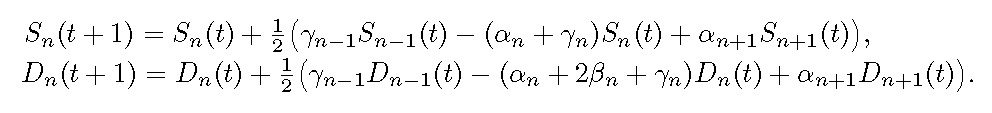


f

Please see the correct equation 12 here: 


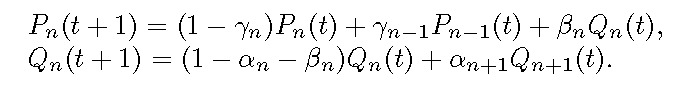


f

Please see the correct equation 13 here: 


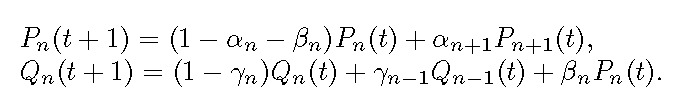


f

Please see the correct equation 18 here: 


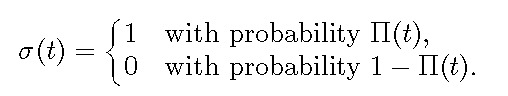


f

